# Monthly Summary

**Published:** 1880-10

**Authors:** 


### MONTHLY SUMMARY.

*Watching the Pulse During the Administration of Chloroform.*—After many years of experience in giving chloroform, both in private practice and as a hospital surgeon, I view with some apprehension the views lately put forward by some of our correspondents, that there is no need to watch the pulse in giving chloroform, only the respiration. In inhaling it myself I observed that about a minute was occupied between the time of inhalation and the maximum effect being produced, so that if the patient's pulse begins to intermit, that time has to elapse between the time that the chloroform is taken from the mouth, *with the effects of the chloroform still deepening in intensity.* Surely, then, it is of importance to note the very faint beginning of such mischief.

I have never seen a death caused by chloroform, but I have seen a few cases in which it was necessary to stop the operation for a time, on account of the effects of the chloroform, which, in these cases, always acted firstly on the heart. In no case have I seen cessation of the respiration. The experience, however, of death by chloroform in the human subject of any one medical man is necessarily so limited, and the circumstances of such deaths are so little favorable for scientific observation, that I think it is highly dangerous to found a mode of practice on the ideas of one or two, who may be inclined to look on the cessation of respiration as the first danger to be avoided, and the only one to be carefully looked for. Perhaps, in regard to this, I may be allowed to speak from my experiments made on animals in 1875–76.

In these experiments, *inter alia*, I found that in cats, when chloroform was rapidly administered (the best way, in my opinion, as avoiding that saturation of the blood with chloroform which is apt to be produced by slow administration, and which renders stoppage of the heart's action, if it does occur, almost surely fatal) the systolic action of the heart *invariably* ceased before respiration. If the chloroform was then removed at once, it was sometimes easy enough to restore animation, but if the chloroform was continued until the respiration stopped also, the chances of recovery were infinitely reduced, and disappear when auricular action ceases. I found on post-mortem examination and experiment that chloroform invariably stopped the action of the heart when found on it.

Wishing to protect the heart's action against the chloroform, I, after several preliminary experiments, used a large, but not a lethal, dose of atrophine, injected about an hour previously to the administration of the chloroform. I now found that the respiration invariably stopped before the heart's action, stoppage of the latter being exceedingly difficult to produce in one case; indeed, I could not kill the cat, though I gave it chloroform in as strong saturations as I possibly could for thirty-five minutes (two minutes being generally sufficient to kill,) and recovery after stoppage of respiration was generally *spontaneous*, respiration recommencing of itself; but when I pushed the chloroform till all signs of heart movement were stopped, the effects were the same as if no atrophine had been given.

Whatever opinions may be held as to the propriety of continuing the use of chloroform as an anæsthetic, it is still used by many every day, and while it continues to be so, it is of importance that all precautions for using it safely should be taken.

In conclusion, I may say that I should be glad to know that any hospital or other surgeon who still continues to use chloroform often was taking advantage of the hints to be gleaned from my experiments as to the use of atrophine as a heart protector during its administration.—*Dr. Munro in British Med. Journal.*

*Use of Chloroform in Diseases of the Heart.*—On this subject M. Vergely, of Bordeaux (*La France Medicale,*) remarks that there is a difference of opinion, some asserting chloroform is very useful, and others that it does harm in affections of the heart. In M. Vergely's memoir, to which M. Dieulafoy has recently drawn the attention of the Societe Medicale des Hopitaux, three principal points are established.

1. That the existence of heart disease does not contraindicate the use of anæsthetics.
2. That chloroform is a sedative in this class of diseases.
3. That it should be used with discretion. In some cases of severe palpitation chloroform may be successfully administered. Also in some cases of dyspnœa and palpitation arising from miteal insufficiency, ether alone or conjointly with hypodermic injections of morphia. M. Vergely has also given it without any accident in angina pectoris, and in certain other affections of the heart characterized by dyspnœa and palpitation. From inquiries he has made into the literature of the subject, he concludes that this agent has been employed too timidly and unsystematically.—*Druggists Circular.*

---

*Tragedy in a Dental Office.*—On July 26th, at about 5 o'clock, a terrible tragedy occurred in Oakland, California, the persons concerned being E. F. Schroeder, exchange teller in the London and San Francisco Bank, and Dr. Alfred Lefevre, a prominent dentist of Oakland. The latter was shot and killed by Schroeder, who visited him in his office, and fired two shots, one of which entered Dr. Lefevre's left side, ranging downward through the intestines and lodging in the opposite hip. The second shot missed, but the right side of the head and ear were powder-burned.

A San Francisco daily states that Mr. Schroeder has refused to make any statement regarding the affair, and the following particulars have been gathered from persons not directly interested.

According to the reports, it appears that Mrs. Schroeder, who is a daughter of Rev. Horatio Stebbins, of San Francisco, went down to the train on the day of the shooting, to meet her husband on his arrival from San Francisco. She was accompanied by her little daughter, some three years of age. Upon meeting her husband, Mrs. Schroeder is represented to have told him that on the Saturday previous, while under the influence of chloroform in Dr. Lefevre's office, a felonious assault had been made upon her by the dentist in question.

After hearing the story, Schroeder went with his wife and child to Dr. Lefevre's office corner of Eighth street and Broadway, and mounting the stairs, entered the Doctor's office. Advancing within a few feet of Lefevrc, he pulled a pistol and fired two shots. One report states that Schroeder said, "Dr. Lefevre, this is my wife; take this," (firing) "and take that" (firing again.) The shooting took place at 4:50 o'clock, and Schroeder was almost immediately arrested. On the way to the City Prison, Schroeder said: "I hope to God I have killed him ; if I haven't I will. A man cannot seduce my wife and live."

Dr. Lefevre, upon being shot, fell on the threshold of the door leading from the operating room to the private working room. He was raised and placed upon a sofa and was soon surrounded by physicians who had been summoned. The wound was fatal, Dr. Lefevre survived about forty minutes.

There are many people who believe thet Mrs. Schroeder's charge against the deceased was purely illusory. It is well known that such hallucinations are not uncommon after the administering of chloroform. Some remarkable cases exist where hallucinations of this nature have taken the form of absolute conviction in the minds of the persons laboring under them, although there exists abundant evidence to prove that this conviction was utterly unfounded.

Dr. Lefevre was quite well known in Western New York, having been a student in the office of the late Dr. J. G. Barbor, of Le Roy, and went to California in 1862. We are informed that he was a native of Canada.

The coroner's jury rendered a verdict charging Schroeder with the murder of Dr Lefevre.—*Dent. Ad.*

---

*Apparent Death as a Result of Asphyxia.*—Medical journals, says the *Medical Press and Circular*, occasionly informs us of wonderful resuscitations brought about by the persistent use of artificial respiration; but two, lately reported to the Academy of Science, Paris, by Dr. Fort, Professor of Anatomy in the Ecole Pratique, are especially noteworthy, and teach us to persevere in any efforts we may make to bring back signs of life. In the case of a child three years old, who had already been placed in his shroud, Dr. Fort commenced the use of artificial respiration three and a half hours after apparent death. After four and a half hours of steady work the child was brought back to life.. The other case was that of a drowned man, who had been under water for twelve minutes before the body was recovered. Artificial respiration was commenced an hour afterward, and after being kept up for hours the man was restored to life. The report of the meeting of the Academy does not give the details of the child's case before the appearance of the apparent death, nor what prompted the doctor to commence the artificial respiration.—*Med. and Surg. Reporter.*

*A New Anæsthetic.*—J. T. Clover, in the *British Medical Journal* writes as follows: "In your last number mention is made of a new anæsthetic, described as a combination of nitrous oxide gas and ethylen dichloride, and introduced in Edinburgh by Mr. Macleve.

I have not used the ethylen dichloride, but I suspect that the substance alluded to is ethidene dichloride, and I have been giving this with laughing gas for the last fifteen months to more than a thousand cases in hospital and private practice. On the whole, I think ethidene dichloride a better anæsthetic than ether or chloroform, and can confirm all that Mr. Macleve says of it; but I have found that when given beyond a certain strength it causes vomiting and depression of the heart's action; and I fear that the plan recommended, of pouring it on a sponge in the tube or supplemental bag, without the means of regulating the strength of the vapor, will produce ere long results less satisfactory. It will be found that half a drachm is not always sufficient; and, if a larger quantity be used and it run through into the inhaler, the dose may be made strong enough to produce serious results. Chloroform applied in the same way would answer very well in the majority of cases; but in an exceptional one it would yield the vapor too abundantly for safety.

I have found a small-sized gas and ether inhaler answer my purpose; but I have no doubt the portable regulating ether inhaler, charged with about two-thirds of the usual supply of ether, would do equally well. In all cases of ethidene inhaling, the pulse should be watched as well as the breathing."

---

*Swallowing False Teeth.*—English papers report an odd case from Carlisle. A doctor was called one evening at 7 o'clock to visit a lady of about 55, who, as she described it, had been feeling poorly all day, but could give no definite symptoms, and complained of no pain. During the examination he noticed a change in her speech. This led to an investigation of her throat. Outwardly the neck appeared normal, and nothing could be felt to indicate an obstruction; he then examined the pharynx, but no foreign body could be seen there, and the examination only brought on vomiting and straining. However, he determined to look a second time, and judge of his surprise to find embedded low back in the pharynx a set of false teeth, which he extracted with little trouble. Upon inquiry, the woman said she had missed her teeth about 9 o'clock in the morning, but had no idea she had swallowed them. It is remarkable that they had been in the pharynx without causing her pain for ten hours.

*Classic Dentistry.*—Dr. Xavier Landerer, of Athens, says in the London *Chemist and Druggist*: "It may be safely asserted that the ancients certainly cleaned their teeth and used tooth powder. If the necessary attention was given, relics would be found in the graves of the women. The word *odontotrimma*, the tooth-scouring stuff or tooth powder, is found in ancient Greek, and in the Greek Pharmacopœia is applied to tooth powder. It is interesting to find that the ancients had made some advance in dentistry. A friend of mine (now dead) occupied himself in collecting ancient Hellenic skulls, wishing to show that they did not differ in shape from those now carried in Greece. Among several hundreds of these skulls, some perhaps 2,000 years old, we found two with 'stopped' teeth. One was filled with a mass as hard as stone, which, on analysis, proved to be hydraulic lime, made from volcanic ash, Santorin earth, and lime. Marvellous as it may seem, the hollow of one tooth in the other skull had been filled with gold thread or gold leaf. The metal used was pure. The skull itself, though deprived of the stopping, is now in the Archæological Museum."

---

*Is Salicylic Acid Injurious to the Teeth?*—The *Journal of Materia Medica* observes that salicylic acid, now so much in use as a dentifrice, is found, by Dr. Buch, of St. Petersburg, to be a solvent, and exceedingly injurious. Dr. Buch states that he was in the habit of using a solution of three parts in one thousand of salicylic acid, a lotion of such strength being fatal to bacteria; in a few weeks, however, he experienced a singular sensation in his mouth; the teeth appeared to become softer, and on the surface something gritty was detected, there being evidently a grandular formation.

Dr. Buch's conclusion is that the substance in question is a salicylate of lime, and, if so, the use of the acid as a dentifrice should be discontinued.—*Medical and Surgical Reporter.*

---

*The Temperature of the Breath.*—A good deal of curiosity is being expressed in regard to the apparent high temperature of the breath, under certain conditions. If we take an ordinary clinical thermometer, wrap it up pretty tightly in several folds of a silk handkerchief, apply it closely to the lips and breathe gently through the silk just over the bulb of the thermometer for about five minutes, always making the inspirations through the nose, we shall find that the thermometer will show a temperature ranging from 101° to 108°, or even 109.5°. Dr. E. S. Clark first called attention to this phenomena in the *Medical Herald.* The facts are corroborated by Dr. Dudgeon in the *Medical Press and Circular.*—*Med. Record.*

